# Niche separation in bacterial communities and activities in porewater, loosely attached, and firmly attached fractions in permeable surface sediments

**DOI:** 10.1093/ismejo/wrae159

**Published:** 2024-08-08

**Authors:** Chyrene Moncada, Carol Arnosti, Jan D Brüwer, Dirk de Beer, Rudolf Amann, Katrin Knittel

**Affiliations:** Department of Molecular Ecology, Max Planck Institute for Marine Microbiology, 28359 Bremen, Germany; Department of Earth, Marine, and Environmental Sciences, University of North Carolina at Chapel Hill, Chapel Hill, NC 27599, United States; Department of Molecular Ecology, Max Planck Institute for Marine Microbiology, 28359 Bremen, Germany; Department of Molecular Ecology, Max Planck Institute for Marine Microbiology, 28359 Bremen, Germany; Department of Molecular Ecology, Max Planck Institute for Marine Microbiology, 28359 Bremen, Germany; Department of Molecular Ecology, Max Planck Institute for Marine Microbiology, 28359 Bremen, Germany

**Keywords:** surface sediments, niches, CARD-FISH, Arctic, 16S rRNA gene sequencing, metagenomes, frequency of dividing cells, hydrolysis rates, oxygen consumption

## Abstract

Heterotrophic microbes are central to organic matter degradation and transformation in marine sediments. Currently, most investigations of benthic microbiomes do not differentiate between processes in the porewater and on the grains and, hence, only show a generalized picture of the community. This limits our understanding of the structure and functions of sediment microbiomes. To address this problem, we fractionated sandy surface sediment microbial communities from a coastal site in Isfjorden, Svalbard, into cells associated with the porewater, loosely attached to grains, and firmly attached to grains; we found dissimilar bacterial communities and metabolic activities in these fractions. Most (84%–89%) of the cells were firmly attached, and this fraction comprised more anaerobes, such as sulfate reducers, than the other fractions. The porewater and loosely attached fractions (3% and 8%–13% of cells, respectively) had more aerobic heterotrophs. These two fractions generally showed a higher frequency of dividing cells, polysaccharide (laminarin) hydrolysis rates, and per-cell O_2_ consumption than the firmly attached cells. Thus, the different fractions occupy distinct niches within surface sediments: the firmly attached fraction is potentially made of cells colonizing areas on the grain that are protected from abrasion, but might be more diffusion-limited for organic matter and electron acceptors. In contrast, the porewater and loosely attached fractions are less resource-limited and have faster growth. Their cell numbers are kept low possibly through abrasion and exposure to grazers. Differences in community composition and activity of these cell fractions point to their distinct roles and contributions to carbon cycling within surface sediments.

## Introduction

Permeable marine sediments are efficient filters for bioavailable compounds from the water column [1, 2]. They cover at least half of the continental margins and are biogeochemically highly active: the majority of sublittoral sediments are permeable sands that allow an advective transport of oxygen and particulate and dissolved organic matter into the sediments [3, 4]. Most organic carbon is quickly remineralized by benthic heterotrophic microbes, reducing the amount of organic matter buried and hence, the CO_2_ removed from the atmosphere [5, 6]. Thus, the activities of heterotrophic microbial communities impact organic matter degradation and elemental cycling in the marine environment across vast spatio-temporal scales [7].

Early investigations of microbial colonization of sand grains found diatoms, algae, and bacteria mostly in protected areas. Abrasion likely prevents microorganisms from colonizing smooth or convex surfaces [8, 9]. More recently, it was found that the smoother surfaces on a sand grain had lower cell densities [10]. Thus, a fraction of cells is securely attached to the grain, whereas others on smoother surfaces may be more susceptible to removal. Although colonizing crevices may provide protection, these sites are limited in advection, so bacterial respiration is limited to the diffusive transport of substrates, oxygen, and other electron acceptors [11]. The physicochemical differences between exposed and protected sites could lead to preferential colonization of specific taxa in different areas on the grains.

Most studies investigating microbial communities in marine sediments characterize the bulk sediment as a whole [12–14]. Only a few studies disentangle the interstitial porewater (PW) community from the community attached to sediment grains. From the few studies that investigated the PW as a separate niche, it was found that this habitat only harbored ~0.2% of the cells living in the sediment [15], and only 2–3% of operational taxonomic units (OTUs) were shared between the overlying water, PW, and the sediment [16]. Similarly, a study of freshwater lakes found that bacterial and viral abundance in the sediments were significantly higher by one to two orders of magnitude than in the PW; only 12%–19% of the OTUs in the PW and sediment overlapped [17]. Additionally, another study analyzed a fine fraction and a coarse fraction of sediments, and found that cells attached to finer particles were more actively respiring than the coarse fraction [15].

Together, these findings emphasize the importance of separately investigating fractions of the bulk sediment in order to understand the role of the different sediment compartments in organic matter degradation and elemental cycling. In this study, we investigate three fractions of benthic bacteria based on their degree of attachment to sediment grains: cells (i) in the PW, (ii) firmly attached to grains (FA), and (iii) loosely attached to grains (LA). We hypothesize that the benthic microbial community in these fractions differs in composition, metabolic capability, and activity: those in the PW and LA fractions are potentially less limited in solutes and oxygen compared with the FA fraction, and thus could harbor more actively respiring cells capable of utilizing high molecular weight organic matter. To test this hypothesis, we designed a sediment fractionation method and tested it on sediments from a polar coastal site. We then combined microscopy, 16S rRNA gene sequencing, metagenomics, polysaccharide hydrolysis rate measurements, and oxygen optode measurements to characterize these fractions in detail. Our findings provide insight into the different bacterial niches and adaptations within permeable surface sediments, where a significant amount of organic matter is remineralized.

## Materials and methods

### Sampling

We collected sediments and overlying seawater (OSW) from Isfjorden, Svalbard (“Station 5”: 78°06′36.0”N, 14°21′00.00″E ± 30 m) using the Ellrott grab [18]. Surface seawater (SSW) was collected using a bucket. Sample collection was carried out on 30 April and 2 May 2022. These samples were used for cell counts, 16S rRNA gene sequencing, CARD-FISH, and metagenomics. In addition, grabs were collected on 27 and 29 April 2023 for cell counts, laminarin hydrolysis rate measurements, and oxygen consumption measurements. Once all OSW had been carefully removed using syringes, we subsampled the surface sediment (0–2 cm depth) for further fractionation. Hereafter, we refer to the topmost 2 cm of the sediment as surface sediments. The oxygen penetration depth at the station was 6.8 mm [18], which means the samples contain both oxic and suboxic or anoxic layers. To have a reference unfractionated sample, we also collected bulk sediment. For both years, the sediment was comprised mostly of fine sand with a fraction of 75 vol% of the grains larger than 122–124 μm and 25 vol% of the grains larger than 230–232 μm (Jürgen Titschack, personal communication). The water depth around the station was 4.5 m and the OSW salinity for both years was 35 PSU. The surface sediment temperature ranged from −0.5 to 0.8°C in 2022 and −0.2 to 0.3°C in 2023.

### Porewater extraction and separation of loosely and firmly attached cells

**Figure 1:**
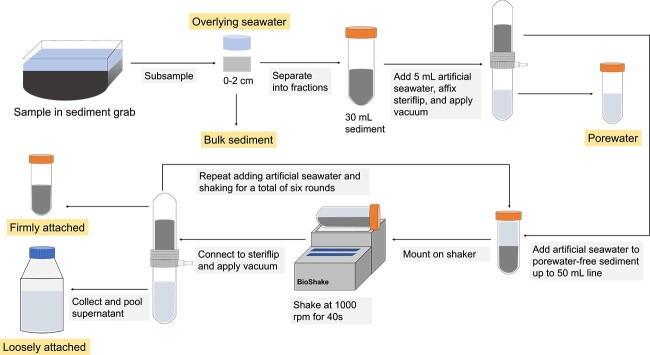
Schematic diagram of the fractionation protocol. First, the PW is extracted from the sediment using a Steriflip filtration unit (60 μm pore size). Then, the LA cells are separated from the firmly attached cells by shaking the grains in ASW on a BioShake iQ mixer at 1000 rpm for 40 s, followed by filtration of the supernatant through a Steriflip filtration unit after each round of shaking. The shaking procedure is repeated for a total of six rounds, and the supernatants are pooled. The firmly attached fraction are the cells remaining on the grains after the six rounds of shaking.

A few hours after collection, sediments were fractionated into the PW, loosely attached (LA), and firmly attached (FA) cells (Fig. 1). To extract the PW, we transferred 30 mL of sediment to a 50 mL tube. Then, 5 mL of sterile artificial seawater (ASW: 450 mM NaCl, 59.6 mM MgCl_2_, 56.5 mM MgSO_4_, 13.2 mM CaCl_2_, 9.7 mM KCl, 0.8 mM KBr, 0.3 mM H_3_BO_3_, 0.1 mM SrCl_2_) was added to wash out the PW and the tube was affixed to a Steriflip filter unit (Merck KGaA, Darmstadt, Germany) with a 60 μm pore size. This pore size was selected based on the minimum grain size at the station in Isfjorden [19], such that grains could not pass through. The tube was then inverted and a vacuum was applied (−600 mbar) to extract the PW from the sediment. The PW fraction contains free-living cells as well as cells attached to particles <60 μm. To obtain the LA fraction, we filled the tube containing the PW-free sediment with ASW up to the 50 mL line, affixed the tube to a BioShake iQ shaker (Q Instruments GmbH, Jena, Germany) and shook the tube for 40 s at 1000 rpm (mixing diameter: 2 mm; linear speed: 10.5 cm s^−1^). The shaking was used to simulate strong sediment reworking events at the site (Supplementary Video 1; compilation of photos taken hourly from the station from 30 April until 3 June 2022). The mean depth-averaged current speeds in Isfjorden were reported to be on average 7 cm s^−1^ with a maximum value of 35 cm s^−1^ [20]. After shaking, we separated the supernatant from the sediment using another 60 μm Steriflip. We repeated the shaking step for a total of six rounds, adding new ASW each time and pooled all supernatants. The Steriflip filtration unit was replaced after two uses to prevent clogging. After six rounds of shaking, cells remaining on the grains were defined as the FA cell fraction.

### DNA extraction, 16S rRNA gene amplification, sequencing, and analysis

To collect cells from the SSW, OSW, PW, and LA fractions, we filtered the samples through a Sterivex filter (pore size 0.22 μm; Merck KGaA, Darmstadt, Germany), and used the membrane for DNA extraction. Due to differences in sample availability, filtered volumes varied across the fractions: 250 mL of SSW, 150 mL of OSW, 7–15 mL of PW, and 30–50 mL of LA were filtered. For FA and bulk sediment, ~250 mg of sediment was used as input. DNA was extracted from all the fractions using the ZymoBIOMICS DNA/RNA Miniprep kit (Zymo Research, CA, USA). To amplify the V3-V4 region of the 16S rRNA gene, primers S-D-Bact-0341-b-S-17 and S-D-Bact-0785-a-A-21 were used [21]. The amplicons were sequenced on a MiSeq (2 × 300 bp; Illumina Inc, CA, USA) at the Max Planck Genome Center in Cologne, Germany. Details on the sequence analyses are described in the supplementary information.

### Cell counts

To count the number of cells in the fractions, we fixed the samples with formaldehyde (final concentration: 1.5%) directly after retrieval and incubated them for 1–4 h at room temperature. Details on the sample processing are described in the supplementary materials and methods. Manual counting was done using a Nikon 50i epifluorescence microscope (Nikon Instruments, Inc, USA). The cell abundance of free-living and particle-attached cells in the PW was also determined manually via epifluorescence microscopy.

### CARD-FISH and measurement of frequency of dividing cells

Catalyzed reporter deposition - fluorescence *in situ* hybridization (CARD-FISH) with horseradish peroxidase-labeled probes was done on all fractions following the protocol described previously [22], with modifications as described in [19]. Probes and formamide concentrations used are given in Table S1. Automated imaging of FISH-stained cells were done as described previously [19]. The images were then analyzed using the Automated Cell Measuring and Enumeration tool 3.0 (ACMEtool) [23]. The frequency of dividing cells for a subset of the taxa, namely *Bacteria*, *Bacteroidota*, *Gammaproteobacteria,* and *Woeseiacae* were determined in the different fractions and seawater using a method described previously [24]. Dividing cells were identified by two intracellular DNA-stain maxima, compared with one maximum in non-dividing cells.

### Analysis of community-level metabolic potential

DNA from fractions and bulk sediment obtained from the same grab were sequenced on a PacBio Sequel IIe platform (Pacific Biosciences, CA, USA) at the Max Planck Genome Center in Cologne, Germany. The goal of the metagenome analysis was to obtain an overview of the carbohydrate-active enzymes (CAZymes) and respiration capabilities in the fractions. Therefore, the analysis was done at the read level, rather than after metagenome assembly. Genes for CAZymes and for aerobic and anaerobic respiration were annotated using several different databases (Table S2, supplementary materials and methods), and the number of hits for each sample was normalized against the sequencing depth of the beta subunit of the bacterial RNA polymerase gene (*rpoB*) for the same sample. Further details on the library preparation and metagenome analyses are provided in the supplementary information.

### Incubations with fluorescently-labeled laminarin and extracellular hydrolysis rate measurements

Laminarin is a diatom- and algal-derived polysaccharide, and its hydrolysis is known to proceed rapidly in seawater and sediments, including those from Svalbard [25]. To measure extracellular hydrolysis rates of laminarin, samples were incubated with fluorescently labeled laminarin (FLA-laminarin) that was synthesized and characterized as described in [26]. Two substrate incubation setups were used (Fig. S1). One setup had all fractions amended with equal concentration of FLA-laminarin (35 μM), and another setup used varying concentrations based on expected cell numbers in a fraction (i.e. approximately equal amounts of FLA-laminarin per cell). For the latter, the final concentration for OSW was 3.5 μM, PW and LA were 35 μM, and FA and bulk had 100 μM FLA-laminarin. In both cases, incubations were carried out in triplicate in the dark at 4°C, along with autoclaved killed controls. Subsamples were taken at the start of the incubation, and after 0.25, 1, 2, 3, 5, 7, and 11 days. At each sampling date, 1 mL of supernatant was sampled with a syringe and filtered through a 0.2 μm filter (SFCA membrane, Thermo Fisher Scientific, MA, USA) and frozen at −20°C until analysis. Hydrolysis rates were measured via changes through time in the molecular weight of the FLA-laminarin as it was progressively hydrolyzed [26]. Calculations for converting rates in the incubations back to nmol monomer L^−1^ sediment h^−1^ or L seawater h^−1^ are detailed in the supplementary information.

### Oxygen consumption measurements via optodes

To compare the rate of oxygen consumption among the fractions, sediments from three grabs were fractionated, and from each grab, at least two replicates for each fraction were measured. Immediately after fractionation, oxygen consumption by the fractions was determined by an oxygen optode (OXF50-OI, Pyroscience GmbH, Aachen, Germany). Prior to measurements, the oxygen optode was 2-point calibrated in oxygen-free sodium ascorbate solution (1 M sodium ascorbate, pH 11) and air-saturated water. The fractions were kept at 2–4°C. The cell suspensions were saturated with air. The FA fraction, still attached to the grains, was resuspended in ASW and then saturated with air. Once the oxygen concentration stabilized, the air supply was stopped, and the decrease in oxygen concentration was measured over time. Data acquisition was done using Profix software (PyroScience GmbH, Aachen, Germany).

## Results

### Cell counts

**Figure 2:**
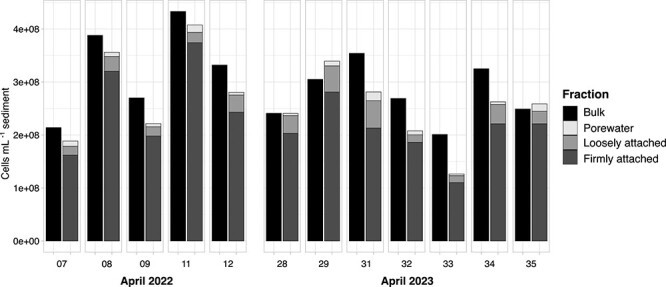
Cell counts in bulk sediments and in the corresponding fractions for April 2022 and April 2023. Each panel represents one biological replicate, with numbers indicating the grab number.

Although bulk sediment cell numbers varied somewhat between grabs, cell numbers on average were similar for both years. The average cell number in the bulk sediment was 3.3 × 10^8^ ± 1.0 × 10^8^ and 2.8 × 10^8^ ± 0.5 × 10^8^ cells mL^−1^ for April 2022 and 2023, respectively (Fig. 2). Most cells were firmly attached, whereas cell counts in the PW and LA fractions were one order of magnitude lower. In the PW, 42 ± 3% of cells were free-living, and 58 ± 3% of cells were attached to small particles of about 5–15 μm diameter (Fig. S2). The fraction of free-living PW bacteria is likely larger; free-living cells that coincidentally ended up on top of a particle cannot be distinguished from cells originally attached *in situ* and are therefore misassigned to the particle-attached fraction. The cell numbers in the OSW were an order of magnitude lower than in PW (Fig. S3).

### Bacterial community composition and diversity in PW, LA, and FA fractions

**Figure 3:**
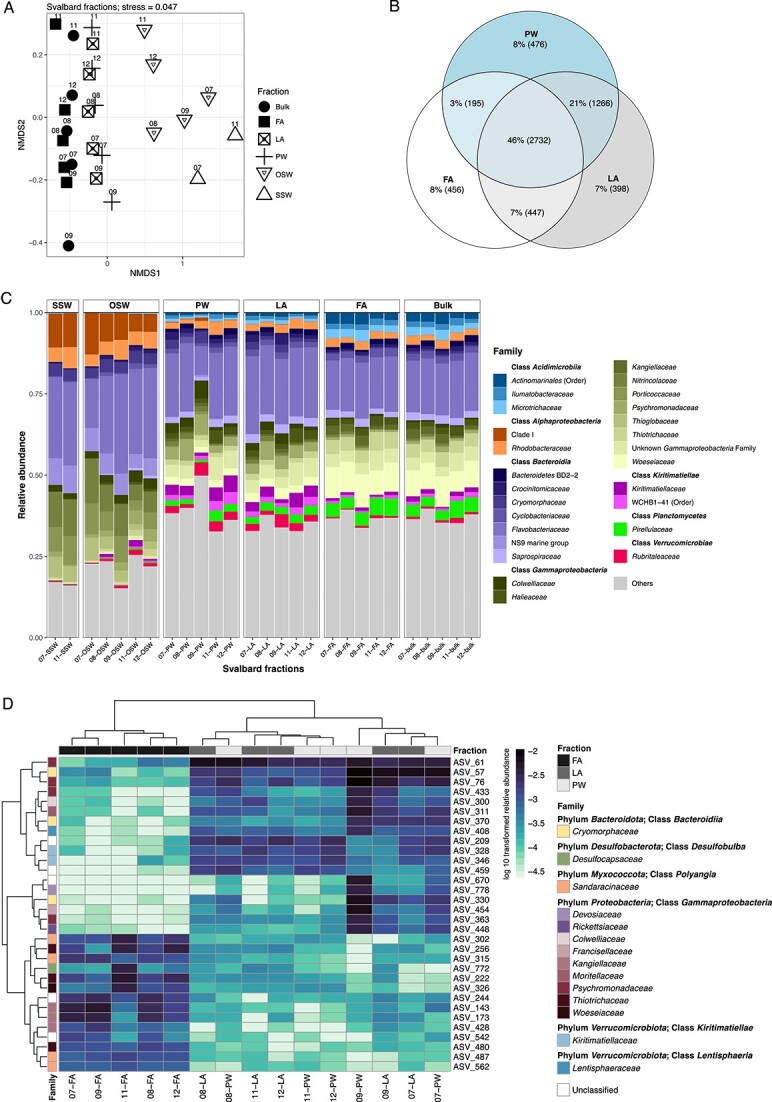
Bacterial community composition based on 16S rRNA genes retrieved from seawater, bulk sediment, and fractions. (A) Non-metric multidimensional scaling (NMDS) plot at the ASV level showing dissimilarities between the samples. Numbers next to symbols indicate the grab number. SSW = surface seawater, OSW = overlying seawater, PW = porewater, LA = loosely attached cells, FA = firmly attached cells. (B) Shared and unique ASVs in the fractions, in raw numbers and percentages. An ASV was considered present if it was detected in 3 out of 5 replicates. (C) Most abundant families (>1% relative abundance) across all samples. The families are grouped and colored by taxonomic class. For taxa which are unclassified at the genus level, the higher taxonomic classification and rank are indicated. (D) Heatmap of ASVs with significant differential abundance (adjusted *P* value < 0.01) determined by DESeq2. Only the ASVs with the 10 highest and 10 lowest fold change in each comparison are shown. Columns are clustered according to sample similarity and rows are clustered according to ASV abundance values. All ASVs with significant differential abundance are enumerated in Table S8.

Bacterial 16S rRNA gene sequence data showed that communities in the seawater differed from all sediment fractions (Fig. 3A and C). We also found lower species richness in the seawater samples compared with the sediment (Table S3). Among the fractions, richness based on the number of observed ASVs and evenness based on the inverse Simpson index were lower in FA compared with PW and LA. Within fractions, PW and LA clustered close to each other whereas bulk samples were positioned in between the FA and the LA and PW samples, representing the expected composite of the fractions (Fig. 3A). The number of shared ASVs in the fractions supported the NMDS clustering (Fig. 3B). Moreover, when comparing the OSW, PW, and LA communities, the PW was different from the OSW (Fig. S4A) and was more similar to LA (Fig. S4B). Analysis of similarity (ANOSIM) confirmed that the OSW was significantly dissimilar to the rest of the fractions (Table S4). For example, SAR11 Clade I was only found in the SSW and OSW samples, but not in the sediment fractions (Fig. 3C). Within the sediments, the PW and LA communities were barely separable, but these two fractions were well separated from FA. Although all fractions were dominated by heterotrophs like *Flavobacteriaceae,* they differed at the ASV (Fig. S4C) and genus level: in the SSW and OSW, *Polaribacter* spp. were more dominant, in PW and LA, *Lutibacter* spp. and *Lutimonas* spp. dominated, and in FA, *Maribacter* spp. were more abundant. Moreover, *Woeseiaceae* and *Acidimicrobiia* (*Ilumatobacteraceae* and *Microtrichaceae*) were especially abundant in FA. ASV relative abundances are given in Table S5. Differences in the community inferred from the metagenomic reads and from 16S rRNA gene sequences extracted from the metagenomes showed similar trends as the amplicon data (Tables S6 and S7).

Differential abundance analysis showed similar ASVs and relative abundances in the PW and LA fractions. In contrast, there was a large dissimilarity between the FA and PW, and between the FA and LA fractions (Table S8). To further filter these ASVs to show the highest differences, we selected 10 ASVs with the highest fold change (Fig. 3D). In PW and LA, ASVs belonging to the genera *Lentisphaera*, *Psychromonas*, and *Moritella* were significantly more abundant compared with FA. In contrast, ASVs with the highest fold change in FA compared with PW or LA were affiliated with *Kangiellaceae*, *Sandaracinaceae*, *Thiotrichaceae*, *Desulfocapsaceae*, *Woeseiaceae*, and *Actinomarinales*.

### *In situ* abundance of major taxa and frequency of dividing cells

**Figure 4:**
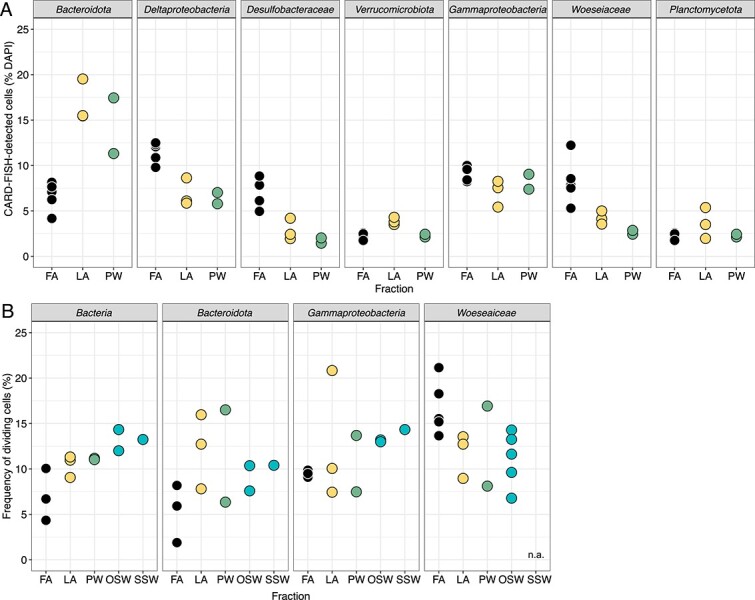
Abundance and frequencies of dividing cells of major taxa. (A) CARD-FISH probe counts relative to total DAPI counts. (B) Frequency of dividing cells obtained from a selection of CARD-FISH microscopy images. n.a.: not analyzed.

Differences in the composition of the fractions were also demonstrated by the *in situ* abundances of major bacterial taxa determined by CARD-FISH (Fig. 4A). *Bacteroidota* abundance was highest in LA and PW, and least abundant in FA. In contrast, FA had the highest abundances of *Deltaproteobacteria*, *Desulfobacteraceae*, and *Woeseiaceae*. In terms of frequency of dividing cells, the data generally showed higher frequencies in the PW and LA fractions compared with the FA fraction, except for *Woeseiaceae* (Fig. 4B). For this taxon, the frequency of dividing cells was highest in the FA fraction.

### Metabolic potential of communities in the fractions

**Figure 5:**
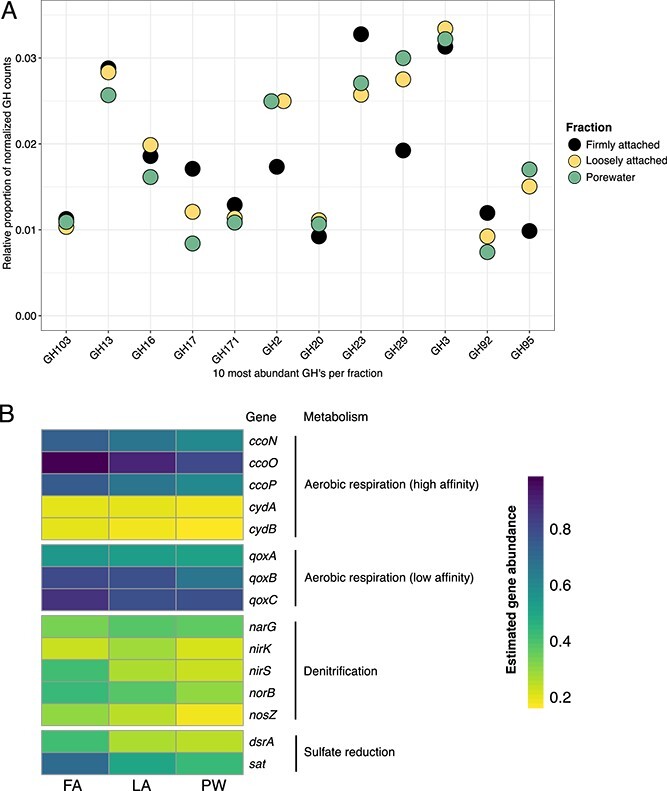
Metabolic potential of bacterial communities in the fractions based on metagenomic read annotations. Gene counts were normalized according to gene length and the coverage of the single copy *rpoB* gene. (A) 10 most abundant GHs in each fraction. *rpoB*-normalized GH counts were additionally transformed into relative abundances with respect to all annotated carbohydrate-active enzymes in each sample. (B) The proportion of the community predicted to encode representative genes for aerobic and anaerobic respiration.

Metagenomic read annotations showed that the PW and LA fraction had similar glycoside hydrolase (GH) and polysaccharide lyase (PL) potential that were different from the FA fraction (Fig. S5). Focusing only on the 10 most abundant GHs per fraction, the fractions possessed similar abundant GHs, but some GHs differed in relative proportion (Fig. 5A). For example, fucosidases GH29 and GH95 had higher proportions in PW and LA. In contrast, the firmly attached fraction had a higher proportion of GH23 (peptidoglycan lyase) compared with both PW and LA.

In addition, key genes for aerobic respiration, denitrification, and sulfate reduction were detected in all fractions, but at varying abundance (Fig. 5B). The genes for sulfate reduction were most abundant in the FA fraction. Denitrification genes did not show this trend, except for the *nirS* (nitrite reductase) and *norB* (nitric oxide reductase) genes. There were no clear differences in the estimated gene abundance of cytochromes, but *ccoO* and *qoxC* (encoding cbb3-type cytochrome-c oxidase subunit II and cytochrome aa3 quinol oxidase subunit III, respectively) had highest read abundance in FA.

### Activity based on extracellular hydrolysis rates of FLA-laminarin and oxygen consumption

**Figure 6:**
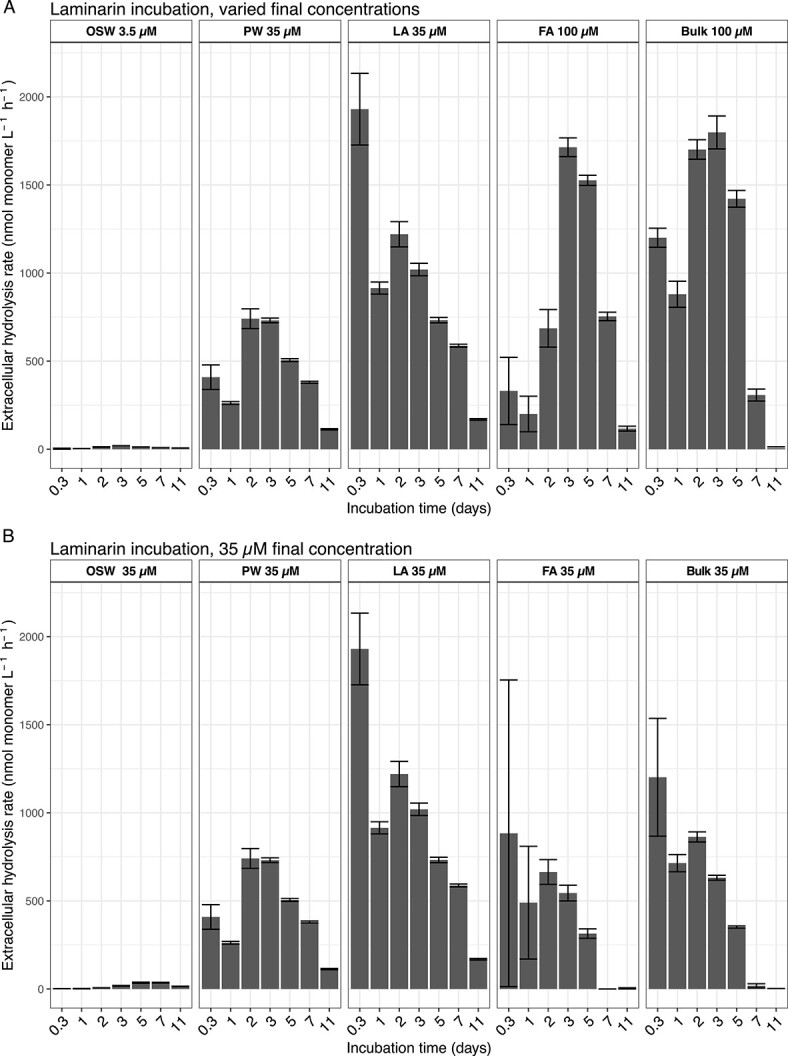
Potential extracellular hydrolysis rates (in nmol monomer equivalent L^−1^ sediment or seawater h^−1^) of laminarin in the OSW, sediment fractions, and bulk sediment. (A) Hydrolysis rates measured from incubations with varying final concentrations of added FLA-laminarin. (B) Rates measured from incubations amended with a final concentration of 35 μM FLA-laminarin.

Extracellular hydrolysis rates of laminarin, a common polysaccharide degraded by marine bacteria [27], differed in the sediment fractions and in seawater. The laminarin hydrolysis rate in the sediment fractions was two orders of magnitude higher than in seawater. The highest laminarin hydrolysis rates were measured in the LA fraction (Fig. 6). The LA fraction responded early and rapidly (1930 nmol monomer equivalent L^−1^ sediment h^−1^ measured after 8 h), whereas high hydrolysis rates in the FA fraction (1714 nmol monomer equivalent L^−1^ sediment h^−1^) were measured only at later timepoints. In addition, comparatively high rates were measured in the PW, considering this fraction has 1–2 orders of magnitude fewer cells than LA and FA, respectively. Maximum rates (741 nmol monomer equivalent L^−1^ sediment h^−1^) were reached after 2 days in the PW incubation.

**Figure 7:**
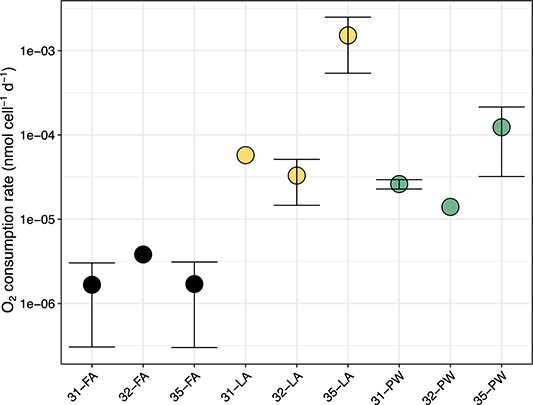
Oxygen consumption rate (in nmol cell^−1^ d^−1^) across sediment fractions. Each dot represents one grab. Error bars indicate the standard deviation from at least three technical replicates. Dots with no error bars represent the mean of two technical replicates.

The LA and PW fractions had much higher per-cell oxygen consumption rates compared with the FA fraction (Fig. 7). Rates in the LA fraction ranged from 5.8 × 10^−5^ to 1.5 × 10^−3^ nmol O_2_ cell^−1^ d^−1^. The PW fraction had O_2_ consumption rates from 1.4 × 10^−5^ to 1.2 × 10^−4^ nmol O_2_ cell^−1^ d^−1^. Rates measured in the FA fraction were at least one order of magnitude lower than in LA and PW (1.7 × 10^−6^ to 3.8 × 10^−6^ nmol O_2_ cell^−1^ d^−1^).

## Discussion

We developed and applied a method to gain higher-resolution information about sediment bacterial communities and their activities. Previous studies investigating PW bacteria [15, 17] extracted the PW by filtering the sediment through a 0.5 μm or a 5 μm filter, which consequently excluded most particle-attached bacteria living in the PW. In contrast, we used a 60 μm pore size filter (smaller than the minimum grain size at the station) to obtain both free-living and particle-attached cells present in the pore space. Cells in the PW made up 3% of cells in Svalbard sediments. This proportion is much higher compared with a previous study that showed that 0.2% of the cells in Middle Atlantic Bight shelf sands were living in the pore space [15].

The high similarity between the PW and LA fractions (Fig. 3A) may represent a “continuum of attachment” of bacteria to sediment grains. We suggest that the LA fraction comprises cells that are either colonizing exposed surfaces, from which they are more easily sloughed off, or employ other less stable mechanisms for attachment. A practical implication of these results is that caution must be taken when processing sediments for cell counts. Vigorous mixing should be avoided, and grains and particles must be allowed to settle before discarding the supernatant when washing off excess fixative.

Most cells in the sediment were firmly attached (Fig. 2). Adhesion to grains provides bacteria with numerous advantages, such as reduced grazing, increased metabolic interactions with other organisms and a more stable environment [28]. Resuspension of sediments can select for populations that are adhered to the grains [15]. However, high cell densities lead to competition for resources. Consumption rates in these sites are also more limited by diffusive transport [11], which may force some microorganisms into inactive states or even toward death [28]. These limitations can be avoided by cells that are LA or living in the PW.

Although the PW and LA fractions were more similar overall to the FA than to the seawater community, the PW, LA, and OSW still shared 25% of bacterial ASVs. This overlap indicates that there is an exchange of LA cells or cells in the pore space into the overlying water column, and vice versa.

### Sediment fractions differ in community composition and metabolic potential

Fractions differed in taxonomy as well as function, as indicated by cell counts, 16S rRNA gene sequences, and metagenomic data (Figs 3, 4A, and 5). PW and LA fractions were distinguished from the FA fraction, as shown by differential abundance analysis yielding indicator ASVs belonging to the genera *Psychromonas*, NS10 marine group, *Lentisphaera*, and *Moritella*. Except for the NS10 marine group, which was mostly found in pelagic samples [29, 30], these taxa have been found in both seawater and marine sediments [31–34]. Apart from *Moritella* spp., they have also been detected in a previous study of bulk sediments in Isfjorden, Svalbard, albeit in lower numbers [19]. Previous studies have shown that these bacteria respond to fresh input of high molecular weight organic matter [29, 30, 35–40]. In the FA fraction, other taxa were dominant. The ASVs with the highest fold change compared with PW or LA belonged to the families *Woeseiaceae*, *Kangiellaceae*, *Sandaracinaceae*, and *Desulfocapsaceae*. The higher abundance of *Woeseiaceae* ASVs in FA versus PW and LA was further supported by higher cell numbers detected by CARD-FISH, and higher frequency of dividing cells in this fraction. Generally, the dominant ASVs in the FA fraction are known to be less dependent on high molecular weight organic matter, but rather on amino acids or fermentation products, and can perform either sulfur oxidation, nitrate, or sulfate reduction [41–46].

The metagenomic annotations further supported the patterns inferred from amplicon data of different substrate niches and respiration potential between the fractions. The significantly higher abundance of taxa capable of utilizing high molecular weight organic matter in the PW and LA fractions correlated with the higher abundance of GH2, GH29, and GH95 in these fractions. GH2 is involved in the hydrolysis of the algal polysaccharide ulvan, and has also been reported in algae-associated *Bacteroidetes* [47, 48]. Moreover, GH29 and GH95 have fucosidase activity and are present in *Verrucomicrobiota* specializing in the degradation of the highly sulfated polysaccharide fucoidan [49, 50]. In accordance with this finding, several *Verrucomicrobiota* ASVs were significantly more abundant in the PW and LA fractions (Table S8). In contrast, GH23 (peptidoglycan lyases) had higher proportions in the FA fraction. In a different study on Svalbard sediment communities, transcripts of this GH family were upregulated during winter, suggesting the recycling of cell wall components when there is no fresh input of organic matter [51]. In addition, there was a stronger signal for sulfate reduction in the FA fraction. *Desulfocapsaceae,* which were significantly more abundant in FA fraction, may have contributed to the higher prevalence of sulfate reduction genes in this fraction.

There was also an overlap in the composition and metabolic potential of the fractions. The presence of similar GHs emphasizes common pathways and capabilities of organic matter recycling, but show that the PW and LA fraction likely respond to fresh input more readily. All fractions also showed the capability for aerobic respiration. The metagenomes were derived from surface sediments, after all; we have previously shown that the oxygen penetration depth at the station was 6.8 mm [18]. Moreover, frequent resuspension and bioturbation (Supplementary Video 1) resupply the sediments with oxygen. Ripple movement was also observed at the site, indicating that at least the upper 2 cm of the sediment is often turned over. This observation means that layers below the measured oxygen penetration depth are not necessarily permanently anoxic, and that the surface layer is well mixed. In this case, major differences in the composition of well-established benthic bacterial communities are not expected between the top 7 mm and the lower 8–20 mm. However, we cannot exclude the possibility that parts of the investigated 2 cm depth layer are permanently anoxic. As a consequence, the suboxic and anoxic layers are also represented in the dataset, which may skew the observed patterns toward anaerobic taxa and metabolisms. In addition, the cells in the PW fraction, in particular those attached to particles, are also exposed to anoxic microniches, and hence these areas would also select for denitrifiers and sulfate reducers. Although the gene abundances described here come from one metagenomic sample per fraction and should be interpreted with caution, these data reveal the different metabolic potentials in the sediment fractions that can be further investigated in the future.

### Porewater and loosely attached bacteria are generally more active than the firmly attached fraction

Despite the PW and LA fractions having lower cell numbers than the FA fraction, our experiments suggest that these fractions contribute significantly to organic matter turnover and oxygen consumption in surface sediments. These characteristics also correlate with cellular growth: for most of the taxa investigated, the PW and LA fractions had higher frequency of dividing cells compared with the FA fraction. For example, the average frequency of dividing cells for *Bacteroidota* was two times higher in the PW and LA fractions than in the FA fraction. Marine representatives of this phylum possess a high diversity of peptidases and GHs in their genomes, demonstrating their specialization for polymer degradation [52]. Strains from this phylum have been shown to increase in abundances during North Sea algal blooms and contribute significantly to laminarin turnover [53]. In contrast, in the FA fraction we measured a higher frequency of cell division for *Woeseiaceae*. This observation is in line with the diverse metabolic capabilities of this clade, with some heterotrophs likely capable of growing on cell wall material, or chemolithotrophs gaining energy via sulfur or hydrogen oxidation [41, 42]. Hence, we showed for a selection of bacterial taxa that they not only differed in abundance between the sediment fractions, but also in cell division activity. This difference is likely driven by redox conditions and/or the availability of their preferred electron donors.

We also measured high extracellular hydrolysis rates in the PW and LA fractions compared with the FA fraction, despite the 1–2 orders of magnitude lower cell numbers in the LA and PW fractions. The high rates in the PW and LA fractions demonstrate the importance of these less abundant fractions in the hydrolysis of organic macromolecules, which is the initial step in organic matter remineralization in marine sediments [54]. Moreover, rates in all sediment fractions, including the PW were orders of magnitude more rapid than the measured rates in the OSW, an observation reflected also in previous studies of Svalbard sediments and the overlying water column [14, 25]. The similarity of the measured rates between the PW and the other sediment fractions further demonstrate that the PW is a habitat much more similar in composition and activity to the sand grain-associated communities rather than the pelagic community. Similar to our findings, experiments from Smeerenburgfjord, Svalbard, have previously shown that enzymatic hydrolysis rates in bulk sediments were up to three orders of magnitude more rapid than in the OSW [25]. Since not all cells are heterotrophic, however, and not all heterotrophs have enzymes that hydrolyze laminarin, no simple scaling factor can be used to convert the measured laminarin hydrolysis rates to per cell rates.

We also observed higher per-cell oxygen consumption rates in the LA and PW fractions compared with the FA fraction. This observation can be explained by the higher prevalence of anaerobes in the FA fraction and that the cells in PW and LA are generally more active and, hence, have higher aerobic respiration rates. A potential source of bias in the rate measurements, however, could be removal during the fractionation step of organic matter along with the LA fraction. Such organic matter might have also served as substrate for the FA fraction. In such a case, separation of organic matter from the FA cells could have affected the measured rates, such that the values may not reflect *in situ* rates. In any case, the measurement of cell activity via frequency of cell division still showed that bacteria in PW and LA were generally more active. This method is free of the bias introduced by the fractionation steps because samples were fixed immediately after fractionation and thus cells had very little time to respond to this change in organic matter availability. Thus, the independent experiments on the fractions demonstrate that the PW and LA cells comprise the metabolically more active fraction in Isfjorden sandy surface sediments. We hypothesize that the LA cells and the cells in the PW are more active because these fractions are specialized and/or primed to readily consume fresh inputs of organic matter. However, we speculate that despite the advantage of enhanced access to organic substrates, the cell numbers in the PW and LA fractions are kept low by mechanisms such as higher exposure to grazing and abrasion, more fluctuation in the surrounding microenvironment, and increased susceptibility to getting flushed out of the sediment. In contrast, the firmly attached fraction, which may be occupying diffusion-limited areas on the sand grain and has higher competition for resources, could have higher cell numbers because of enhanced protection from abrasion, less accessibility to predators within the sand grain’s depressions, and their surrounding environment may be less variable. In addition, the higher cell numbers could mean low cell-to-cell distances in the FA fraction. Close proximity to other cells could offer both advantages and disadvantages. Quorum sensing might be used to coordinate enzyme production [55], but cells could also compete for resources and secrete secondary metabolites to outcompete their neighbors. As has been remarked previously [11], bacteria in sediments face a predicament: they must protect themselves from mechanical abrasion and at the same time occupy a surface with adequate PW flow.

## Conclusion

By fractionating cells in the uppermost 2 cm of permeable sediments and assessing their composition and metabolic activities, we showed that sediment microorganisms occupy different niches within sediments. The PW and LA cells, which made up 3% and 8–13% of cells, respectively, were enriched in taxa that are responsive to fresh input of organic matter. These two cell fractions are specialized in rapid aerobic remineralization of organic matter, hydrolyzing laminarin at rates comparable to the firmly attached fraction (84%–89% of total cells) despite the 1–2 orders of magnitude lower cell numbers. In addition, we found higher frequencies of cell division and faster per-cell O_2_ consumption in the PW and LA cells compared with the FA fraction. From these observations, we hypothesize that the different sediment compartments offer advantages and trade-offs. Those living in the PW and those LA to grains are less limited by diffusive flow and can easily access electron donors and acceptors through advective transport. However, microbes in the pore space are more exposed to grazers. In contrast, we hypothesize that firmly attached cells, which could preferentially colonize local depressions, are more protected from abrasion and grazing, but high cell densities in these diffusion-limited areas can quickly lead to resource limitation. Therefore, bacteria in this fraction must be able to utilize not just fresh organic matter but also hydrolysis and fermentation products. Because oxygen might also become limited, a part of this fraction must be capable of anaerobic respiration. Overall, our findings indicate that degrees of attachment to sediment grains are not stochastic but are influenced by the surrounding microenvironment and taxa-dependent factors. These cell fractions have distinct roles and contributions to organic matter remineralization within surface sediments.

## Supplementary Material

Revised-Supplementary_information_no_markup_wrae159

Revised-Stab_Niche-separation-in-permeable-sediments_wrae159

Supplementary_video1_wrae159

## Data Availability

Sequences have been deposited to the European Nucleotide Archive (ENA) under accession numbers PRJEB67636 (amplicon data), PRJEB75522 (metagenomic reads from fractions) and SAMEA115040714 (metagenomic reads from bulk sediment).
