# Supplementary material for: Niche separation in bacterial communities and activities in porewater, loosely attached, and firmly attached fractions in permeable surface sediments
Source: ISME J. 2024 Aug 8;18(1):wrae159. doi: 10.1093/ismejo/wrae159 (PMC11368169; doi:10.1093/ismejo/wrae159)
Supplement: Revised-Supplementary_information_no_markup_wrae159 [file revised-supplementary_information_no_markup_wrae159.docx]

**Supplementary information**

**Niche separation in bacterial communities and activities in porewater, loosely attached, and firmly attached fractions in permeable surface sediments**

Chyrene Moncada, Carol Arnosti, Jan D. Brüwer, Dirk de Beer,

Rudolf Amann, Katrin Knittel

**Supplementary materials and methods**

**Processing fixed samples for cell counts**

Fixed bulk sediments and FA fractions were carefully washed three times with a 1:1 mixture of 1x phosphate-buffered saline (1X PBS: 13.7 mM NaCl, 0.27 mM KCl, 1 mM Na_2_HPO_4_, 0.2 mM KH_2_PO_4_, pH 7.2) and absolute ethanol (1:1) to remove excess formaldehyde, allowing the grains and particles to settle (approximately 30 seconds) before discarding the supernatant. To remove the cells from the grains, we sonicated the sediments as described previously [1]. Briefly, formaldehyde-fixed sediments were sonicated for 6 rounds at 86% amplitude, 0.2 s pulses, 30 s using the Sonopuls Mini20 (Bandelin, BANDELIN Electronic GmbH & Co. KG, Berlin, Germany). After six sonication steps, ca. 90% of total cells are recovered. It was found that sonicating for 7-12 more steps recovers most cells, but cells were often damaged [1]. Hence, the cell numbers obtained after six sonication steps may still be slightly underestimated. All samples were filtered onto a 0.2 µm polycarbonate filter (Merck, Darmstadt, Germany). Filter sections were stained with 4’,6-diamidino-2-phenylindole (DAPI, 1 μg mL^-1^ final concentration) in a Citifluor/VectaShield (3:1) mixture (Citifluor: CitiFluor Ltd., London, UK; Vectashield: Vector Laboratories, CA, USA).

**Diversity analyses based on 16S rRNA gene sequences**

We removed barcodes and primers from the reads using cutadapt v1.15 [2] and filtered and quality trimmed the reads using the filterAndTrim function of DADA2 v1.16.0 [3], discarding reads shorter than 100 bp. DADA2 was used to infer amplicon sequence variants (ASVs) with the default settings, but with the pool parameter set to TRUE. We then assigned taxonomy to the ASVs using the SILVA database NR99 v138.1 [4] and assigned species using the SILVA species assignment v138.1. Prior to diversity analyses, absolute singletons were removed from the dataset. Community analyses were done in R v4.2.1 [5]. Alpha diversity indices for the samples were calculated using repeated (n = 100) random subsampling of the data to the minimum number of sequences at the site (18586 sequences) through the subsamplingNGS.R function (https://github.com/chassenr/NGS/blob/master/Plotting/SubsampleNGS.R). To assess differences in community composition between fractions, we used the ordinate function in the phyloseq R package [6] to calculate Bray–Curtis dissimilarities and subsequently produce a non-metric multidimensional scaling (NMDS) plot. To obtain the number of shared and unique ASVs in the fractions, we used the ps_venn function in the MicEco package (https://github.com/Russel88/MicEco). An ASV was considered present in a fraction if it was found in three out of five replicates. The dissimilarity between and within fractions was quantified using an analysis of similarity (ANOSIM) with the R package vegan v2.6.4 [7]. Differentially abundant ASVs between fractions were identified using the DESeq2 R package [8]. For each comparison, only the 400 most abundant ASVs in the fractions being investigated were considered. An ASV was considered to have significant differential abundance when the Benjamini-Hochberg adjusted *P*value was less than 0.01.

**DNA extraction and library preparation for PacBio sequencing of metagenomic reads**

DNA from the porewater and loosely attached fractions were extracted using the ZymoBIOMICS DNA/RNA Miniprep kit (Zymo Research, California, USA). DNA from the firmly attached fraction and bulk sediment was extracted using a modified protocol [9] with three additional freeze-thaw cycles in liquid N_2_ and a 65°C water bath. The extracted DNA from this protocol was further purified using the Zymo-Spin III-HRC filters from the ZymoBIOMICS DNA/RNA Miniprep kit. Isolated DNA was quality assessed by Agilent FEMTOpulse. An ultra-low input library was prepared for the PW, FA, and bulk samples according to the protocol of the manufacturer ("Procedure & Checklist - Preparing HiFi SMRTbell® Libraries from Ultra-Low DNA Input", Pacific Biosciences, Menlo Park, CA, USA). A low input library for the LA sample was prepared following the "Procedure & Checklist - Preparing HiFi Libraries from Low DNA Input Using SMRTbell® Express Template Prep Kit 2.0". Next, a library complex was prepared with "Sequel® II Binding Kit 2.2" and sequenced on a PacBio Sequel IIe device at Max Planck Genome Centre Cologne with “Sequel® II Sequencing Plate 2.0” for 30 hours followed by HiFi (>=Q20) generation on the device.

**Read-level metagenome analyses**

FragGeneScan v1.31 [10] was used to predict genes from the long reads using the default settings, except the error model used was sanger_5 (-train = sanger_5), and the parameter for complete genomic sequences was used (-complete = 1). These settings were previously found to perform optimally for unassembled PacBio HiFi reads, resulting in a high correspondence between the length of predicted proteins and the best match in UniProt TrEMBL [11]. For CAZyme analysis, the annotations were done using both HMMer v3.3.2 [12] and diamond BLASTp v2.1.8.162 [13] against the dbCAN HMMdb v12 [14] and CAZyDB.07262023 databases, respectively [14]. BLASTp hits were filtered based on percent identity > 40% and alignment length > 50%, and HMMer matches were filtered based on an e-value < 1e-15. Only proteins annotated with the same CAZyme by both HMMer and BLASTp were used [15]. To estimate the abundance of genes involved in aerobic and anaerobic respiration, genes for O2 consumption, denitrification, and sulfate reduction were also annotated via HMMer v3.3.2 using specific TIGRFAM, PFAM, and custom Hidden Markov Models (see Table S2 for specific HMMs used). To normalize the sequencing depth of each CAZyme and protein family from every sample, the sequencing depth of the beta subunit of the bacterial RNA polymerase gene (*rpoB*) for the same sample was used. To annotate the predicted genes with *rpoB*, diamond BLASTp was used to search against a previously published *rpoB* database [16]. Additionally, the community composition was characterized from the metagenomic reads using both barrnap 0.7 [17] and kaiju v1.7.3 [18]. The extracted 16 rRNA reads from barrnap were then submitted to the SILVAngs analysis pipeline [4]. For assigning taxonomy to reads via kaiju, we used the NCBI BLAST non-redundant database containing all proteins belonging to archaea, bacteria, viruses, fungi, and microbial eukaryotes (kaiju_db_nr_euk_2023-05-10).

**Calculations of hydrolysis rates in the incubations back to nmol L sediment^-1^ h^-1^**

Bulk sediment incubation

Set-up:

- 3 mL bulk sediment (with porewater) + 10 mL FLA-laminarin/ artificial seawater
- Porosity of the sediment is 0.42

Therefore, to calculate the rate back to per L sediment:

$$\frac{Rate x nmol}{L incubation\cdot h}\times\frac{L_{incubation}}{L_{sediment}}$$

( 1 )

$$= \frac{x nmol}{L inc\cdot h}\times\frac{0.01 L_{ASW}+\left( 0.003 L_{sed}\times0.42 \right)porewater}{0.003 L_{sed}}$$

( 2 )

$$=rate \times3.753$$

( 3 )

Firmly attached incubation

Set-up:

- 3 mL firmly attached fraction + 10 mL FLA-laminarin/ artificial seawater

Therefore, to calculate the rate back to per L sediment:

$$\frac{x nmol}{L incubation\cdot h}\times\frac{L_{incubation}}{L_{sediment}}$$

( 4 )

$$= \frac{x nmol}{L inc\cdot h}\times\frac{0.01 L_{ASW}}{0.003 L_{sed}}$$

( 5 )

$$=rate \times3.33$$

( 6 )

Porewater (PW) incubation

Set-up:

- Original sediment volume: 257 mL (divided into 8 tubes), porosity 0.42
- Per tube, 5 mL artificial seawater was added to overcome capillary forces. Total added ASW (5mL x 8 tubes): 40 mL
- Porewater obtained: 90 mL
- The extracted porewater was filled up to 550 mL to have sufficient volume for the incubations (more volume than what is required for the incubation described in this study, since other incubations were done with the porewater fraction which are not described here)

Therefore, to calculate the rates back to per L sediment, we need a series of dilution factors.

DF1 – dilution of the porewater from the addition of artificial seawater

$$=\frac{Total volume of porewater+ASW}{Expected porewater volume}$$

( 7 )

$$=\frac{\left( 0.257 L\times0.42 \right)+.040 L}{0.257 L\times0.42}$$

( 8 )

$$=\frac{0.14794 L_{slurry porewater}}{0.10794 L_{expected}}$$

( 9 )

DF2 – Dilution of the extracted porewater to 0.550 L

$$=\frac{0.550 L_{diluted slurry porewater}}{0.09 L_{slurry porewater}}$$

( 10 )

DF3 – converting the rate in the porewater to rate per L sediment (based on porosity)

$$=\frac{0.42 L_{porewater}}{1 L_{sediment}}$$

( 11 )

Therefore, the final factor is:

$$\frac{x nmol}{{L incubation}_{diluted slurry PW}\cdot h}\times\frac{L_{diluted slurry PW}}{L_{slurry PW}}\times\frac{L_{slurry PW}}{L_{PW}}\times\frac{L_{PW}}{L_{sediment}}$$

( 12 )

$$= \frac{x nmol}{{L incubation}_{diluted slurry PW}\cdot h}\times\frac{{0.550 L}_{diluted slurry PW}}{{0.09 L}_{slurry PW}}\times\frac{{0.14794 L}_{slurry PW}}{{0.10794 L}_{PW}}\times\frac{{0.42 L}_{PW}}{{1 L}_{sediment}}$$

( 13 )

$$=rate \times3.517$$

( 14 )

Loosely attached incubation

Set-up:

- Obtained 619 mL loosely attached fraction from 125 mL sediment (4 x ca. 30 mL sediment, 6 rounds of shaking per tube).

Therefore, to calculate the rate back to per L sediment:

$$\frac{x nmol}{L_{loosely attached fraction}\cdot h}\times\frac{{0.619 L}_{loosely attached fraction}}{0.125 L_{sediment}}$$

( 15 )

$$=rate \times4.952$$

( 16 )

**Supplementary Figures**

|  |
| --- |
|  |

Figure S1. Substrate incubation set up with fluorescently-labelled laminarin.

|  |
| --- |
|  |

Figure S2. Laser scanning micrographs of DAPI-stained cells on a sand grain and in the fractions. A. Cells on an unfractionated bulk sample. B. Cells remaining on a sand grain after removing loosely attached cells (= FA fraction). C. Loosely attached cells. D. Cells in the porewater.

|  |
| --- |

| 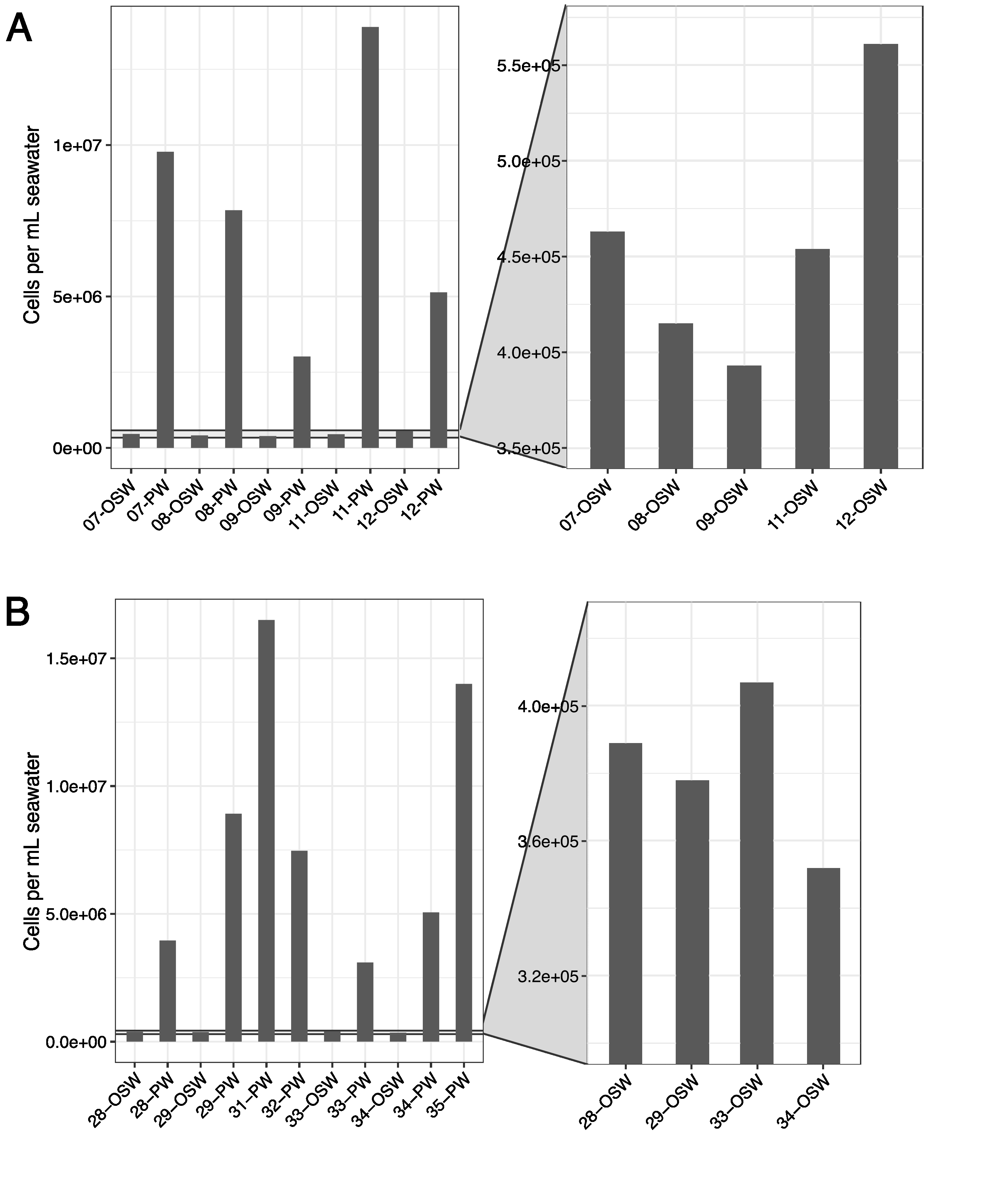 |
| --- |
|  |

Figure S3. Cell counts in seawater and porewater in Svalbard samples for A) April 2022 and B) April 2023. Inset shows a zoomed-in plot of only the overlying seawater samples.

|  |
| --- |
|  |

Figure S4. ASV-level differences in the fractions. A. Shared and unique ASVs in the overlying seawater and porewater. B. Shared and unique ASVs in the overlying seawater, porewater, and loosely attached fraction. For both A and B, an ASV was considered present if it was detected in 3 out of 5 replicates. C. 50 most abundant ASVs in the fractions. Each bar represents one replicate grab. ASVs are grouped and colored according to taxonomic order. When possible, the species name is indicated. Otherwise, the next higher taxonomic classification and rank are indicated.

|  |
| --- |
|  |

Figure S5. Clustered heatmaps of the carbohydrate-active enzyme potentials of the communities in Svalbard bulk sediment and fractions annotated from long metagenomic reads. Relative proportions were centered log ratio transformed. A. Glycoside hydrolase (GH) annotations. B.) Polysaccharide lyase (PL) annotations. GH’s involved in laminarin degradation are marked with the green asterisks.

**References**

1. Miksch S, Meiners M, Meyerdierks A, et al. Bacterial communities in temperate and polar coastal sands are seasonally stable. *ISME Commun* 2021;**1**(1):29. doi: <https://doi.org/10.1038/s43705-021-00028-w>.

2. Martin M. Cutadapt removes adapter sequences from high-throughput sequencing reads. *2011* 2011;**17**(1):3. doi: <https://doi.org/10.14806/ej.17.1.200>.

3. Callahan BJ, McMurdie PJ, Rosen MJ, et al. DADA2: High-resolution sample inference from Illumina amplicon data. *Nat Methods* 2016;**13**(7):581-583. doi: <https://doi.org/10.1038/nmeth.3869>.

4. Quast C, Pruesse E, Yilmaz P, et al. The SILVA ribosomal RNA gene database project: improved data processing and web-based tools. *Nucleic Acids Res* 2013;**41**(D1):D590-D596. doi: <https://doi.org/10.1093/nar/gks1219>.

5. R Core Team. R: A language and environment for statistical computing. R Foundation for Statistical Computing, Vienna, Austria, 2022.

6. McMurdie PJ, Holmes S. phyloseq: An R Package for Reproducible Interactive Analysis and Graphics of Microbiome Census Data. *PLoS One* 2013;**8**(4):e61217. doi: <https://doi.org/10.1371/journal.pone.0061217>.

7. Oksanen J, Simpson G, Blanchet F, et al. vegan: Community Ecology Package. R package version 2.6-4. 2022.

8. Love MI, Huber W, Anders S. Moderated estimation of fold change and dispersion for RNA-seq data with DESeq2. *Genome Biol* 2014;**15**(12):550. doi: <https://doi.org/10.1186/s13059-014-0550-8>.

9. Zhou J, Bruns MA, Tiedje J. DNA recovery from soils of diverse composition. *Appl Environ Microbiol* 1996;**62**(2):316-322. doi: <https://doi.org/10.1128/aem.62.2.316-322.1996>.

10. Rho M, Tang H, Ye Y. FragGeneScan: predicting genes in short and error-prone reads. *Nucleic Acids Res* 2010;**38**(20):e191-e191. doi: <https://doi.org/10.1093/nar/gkq747>.

11. Orellana LH, Krüger K, Sidhu C, et al. Comparing genomes recovered from time-series metagenomes using long- and short-read sequencing technologies. *Microbiome* 2023;**11**(1):105. doi: <https://doi.org/10.1186/s40168-023-01557-3>.

12. Eddy SR. Accelerated Profile HMM Searches. *PLOS Computational Biology* 2011;**7**(10):e1002195. doi: <https://doi.org/10.1371/journal.pcbi.1002195>.

13. Buchfink B, Reuter K, Drost H-G. Sensitive protein alignments at tree-of-life scale using DIAMOND. *Nat Methods* 2021;**18**(4):366-368. doi: <https://doi.org/10.1038/s41592-021-01101-x>.

14. Zheng J, Ge Q, Yan Y, et al. dbCAN3: automated carbohydrate-active enzyme and substrate annotation. *Nucleic Acids Res* 2023;**51**(W1):W115-W121. doi: <https://doi.org/10.1093/nar/gkad328>.

15. Zhang H, Yohe T, Huang L, et al. dbCAN2: a meta server for automated carbohydrate-active enzyme annotation. *Nucleic Acids Res* 2018;**46**(W1):W95-W101. doi: <https://doi.org/10.1093/nar/gky418>.

16. Orellana LH, Rodriguez RL, Konstantinidis KT. ROCker: accurate detection and quantification of target genes in short-read metagenomic data sets by modeling sliding-window bitscores. *Nucleic Acids Res* 2017;**45**(3):e14. doi: <https://doi.org/10.1093/nar/gkw900>.

17. Seeman T. barrnap 0.7 : rapid ribosomal RNA prediction. 2013.

18. Menzel P, Ng KL, Krogh A. Fast and sensitive taxonomic classification for metagenomics with Kaiju. *Nat Commun* 2016;**7**:11257-11257. doi: <https://doi.org/10.1038/ncomms11257>.
